# Experimental study on the engineering performance of LSP stabilized expansive soil under cyclic drying-wetting condition

**DOI:** 10.1371/journal.pone.0358749

**Published:** 2026-09-21

**Authors:** Lin Qin, Jie Li, Fusheng Zha, Long Xu, Bo Kang

**Affiliations:** 1 School of Intelligent Construction and Transportation Engineering, Hefei University, Hefei, China; 2 China Jikan Research Institute of Engineering Investigations and Design, Co., Ltd., Xi’an, China; 3 School of Resource and Environmental Engineering, Hefei University of Technology, Hefei, China; Erbil Polytechnic University, IRAQ

## Abstract

Conventional binders to treat expansive soil commonly present challenges of high cost and significant environmental burdens. To address these limitations, this study proposes an environmentally sustainable binder termed LSP (lime-sodium silicate amended phosphogypsum) based on the valorization of phosphogypsum (PG). Systematic laboratory tests were conducted to evaluate the engineering performance of LSP stabilized soil under drying-wetting cycles. Results indicate that the expansive soil stabilized with LSP obtains a higher maximum dry density and a lower optimum moisture content. The swelling-shrink potential is weakened as well. However, the successive D-W cycles imposes detrimental impacts on the development of all the geotechnical indexes, including free swelling index, unconfined compressive strength (UCS), shear strength, compressibility and permeability. LSP reduces FSI from 58.6% to 32.1%; UCS decreases by 26.5% after 15 cycles; permeability increases 1.65-fold. XRD and MIP analyses reveal that physical filling by PG powder constitutes the primary mechanism for the improvement of soil engineering properties, while micro-crack development is the controlled mechanism for the deterioration induced by D-W cycles.

## 1. Introduction

Expansive soil is widely distributed globally, which is characterized by significant swelling and shrinkage [1,2]. Seasonal moisture variation induce repeated swelling-shrinkage cycles in these soils, often leading to foundation deformation and slope instability in engineering projects [3–7].

In order to improve the engineering performance and eliminate the potential risk of expansive soil, chemical stabilization using lime, cement and fly ash have been extensively studied and applied [8–10]. However, the treated soils based on the conventional hydraulic binders have poorly long-term durability, especially for that experiencing repeated drying-wetting cycles [11–13]. Besides, utilization of cement and lime also faces environmental challenges, such as high carbon emissions and significant energy consumption [14–17]. Therefore, exploring cost-effective, sustainable and eco-friendly alternatives for the improvement of expansive soil has been attracting more and more attentions of the researchers [18–20]. The rapid development of urbanization and industrialization has caused a year-on-year increase in the output and stockpile of solid waste worldwide. Numerous studies have demonstrated the efficacy of industrial solid wastes, including fly ash, steel slag, blast furnace slag and coal gangue, as alternative binders for enhancing the engineering properties of soils [20–24]. Additionally, the national standard GB/T 51003 (2014) [25] has also made corresponding regulations on the application technical indicators of the abovementioned solid wastes. On this basis, this approach represents a sustainable solution, offering dual environmental benefits through waste valorization and soil stabilization.

Phoshpogypsum (PG), a by-product of wet-process phosphoric acid production, consists primarily of dihydrate gypsum (CaSO_4_ ∙ 2H_2_O; ~ 80 wt%) with minor amounts of harmful impurities. Current PG recycling strategies predominantly target its use in building materials, modified additives and subgrade backfill [26–29]. Among these, utilization in road construction represents the most effective pathway for large-scale PG consumption. Nevertheless, PG recycling as subgrade filler accounts for only 3.1% of the total recycled volumes and remains underutilized [26,30]. Key barriers to broader application in highway/road base layers include poor water stability and the presence of detrimental constituents [31–33]. To enhance the water stability of PG and immobilize impurities, incorporating PG with cementitious materials has been proposed for soil stabilization in road construction [34–36]. However, research on PG-cementitious soil stabilizers still remains at the experimental stage, and their conclusions are often controversial. Systematic studies are lacking regarding PG’s role in diverse stabilization matrices and the underlying modification mechanisms.

The proposed LSP binder differs from conventional PG-lime or PG-cement systems in three key aspects: (1) sodium silicate acts as an alkaline activator to suppress phosphogypsum dissolution; (2) stabilization is dominated by physical filling and cation exchange rather than cement hydration; (3) LSP provides lower carbon footprint and better durability under cyclic drying-wetting conditions. Recent studies on sustainable binders support this direction [37–39].

PG has advantages of low cost, waste valorization and high calcium content, but limitations including water solubility, impurity leaching and low strength when used alone [37,40]. Applied studies confirm the practical value of waste-based stabilization in engineering projects [41].

On this basis, this paper systematically investigates the engineering performance of expansive soil stabilized with lime-sodium silicate amended PG (LSP) under cyclic drying-wetting conditions. Furthermore, the stabilization mechanisms associated to LSP and the deterioration mechanism induced by drying-wetting cycles are examined in depth using advanced microstructural characterization techniques, including X-ray diffraction (XRD) and mercury intrusion porosimetry (MIP).

The unique contributions of this study are: (1) development of a novel LSP binder for full phosphogypsum valorization; (2) systematic durability data of stabilized soil under long-term drying-wetting cycles; (3) establishment of a microscale mechanism linking PG dissolution and microcrack evolution to performance deterioration. The findings support the application of LSP in subgrade and slope stabilization in expansive soil regions.

## 2. Materials and methods

### 2.1 Testing materials

#### (1) Expansive soil.

**Fig 1:**
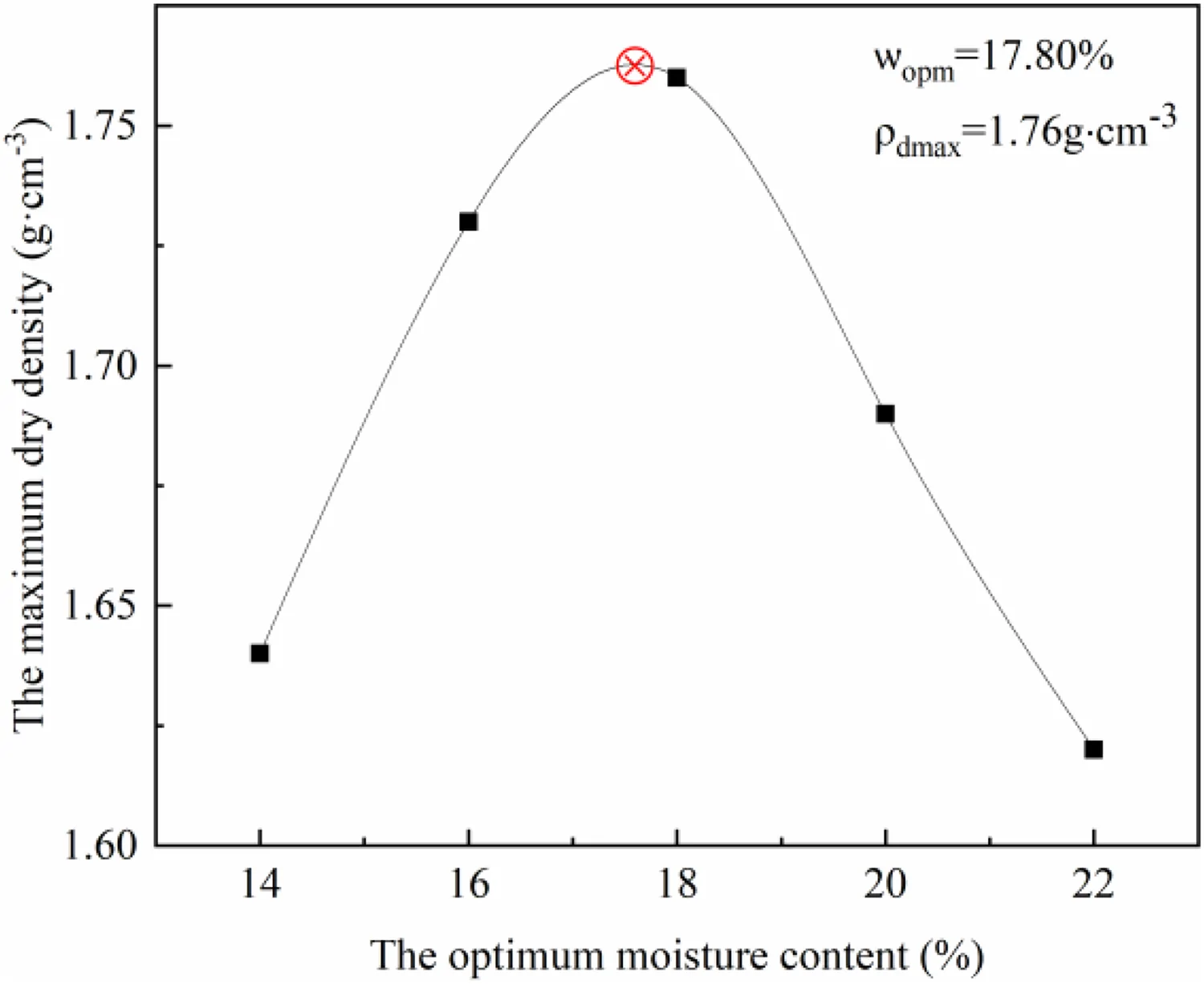
The compaction curve of natural expansive soil.

**Fig 2:**
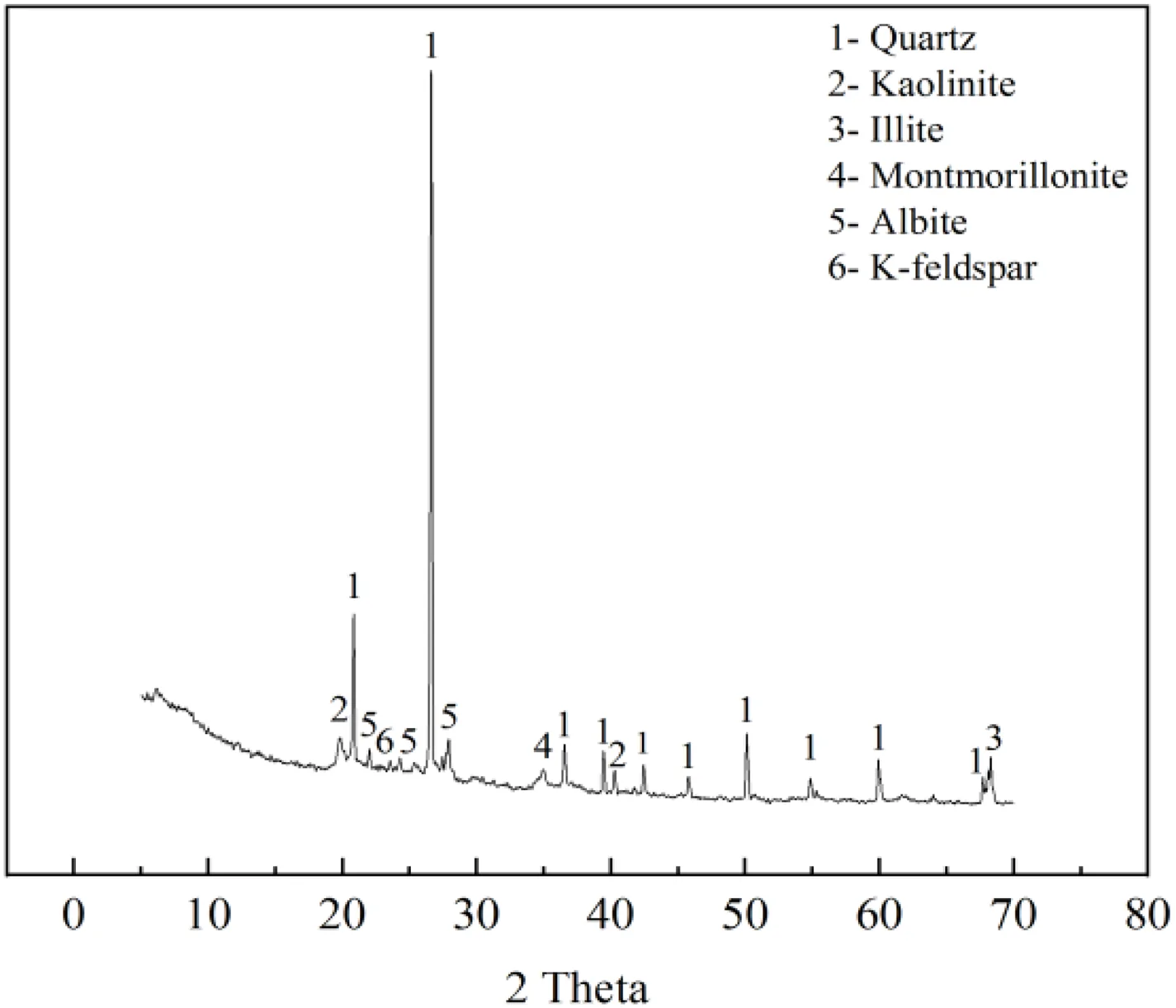
Main mineral composition of expansive soil.

The expansive soil was sampled from a construction site with an excavation depth of 3–4 m in Huainan City, China. No formal permit was required for soil sampling at the construction site in Huainan City, China, as the area was publicly accessible and sampling complied with local environmental regulations. The compaction curve of natural expansive soil is shown in Fig 1. Basic physical properties of the expansive soil were determined in accordance with the Chinese National Standard GB/T 50123 (2019) [42] and are shown in Table 1. The liquid limit and plasticity were 45.1% and 23.1, respectively, with a free swelling index of 58.6%. Based on classification criteria outlined in GB/T 50145 (2007) [43] and GB 50112 (2013) [44], the testing soil is classified as low liquid limit clay (CL) exhibiting slight expansibility. Montmorillonite content is 12.3% (XRD semi-quantitative); differential swell index (DSI) is 28.7%. Soil cation exchange capacity (CEC) = 18.6 cmol/kg. The soil meets Chinese expansive soil criteria: FSI = 58.6% (slight-moderate), plasticity index = 23.1, and contains montmorillonite/illite. The main mineral composition is shown in Fig 2. X-ray diffraction (XRD) analysis indicated that the tested soil is mainly composed of quartz, kaolinite, illite, montmorillonite, albite and K-feldspar. The presence of illite and montmorillonite endows the soil with characteristic of swelling-shrinkage.

**Table 1:** Basic physical properties of expansive soil and phosphogypsum.

Properties	Expansive soil	Phosphogypsum
Liquid limit (%)	45.1	24.0
Plastic limit (%)	22.0	14.3
Plasticity index	23.1	9.7
Specific gravity	2.73	2.15
Water content (%)	24.6	11.45
Maximum dry density (g/cm^3^)	1.76	1.37
Optimal water content (%)	17.8	20.0
Free swelling index (%)	58.6	/
Montmorillonite content (%)	12.3	/
Differential swell index (%)	28.7	/

#### (2) Constituents of the prepared soil binder.

Phosphogypsum (PG) was sourced from a phosphoric acid production facility in Feidong County, Hefei City, China. The initial moisture content was 11.45%, suggesting some degree of aging. Characterization according to Chinese National Standard GB/T 50145 (2007) [43] classified the PG as a low liquid limit silt, with a liquid limit of 24.0% and plasticity index of 9.7 as presented in Table 1. Standard proctor compaction test yielded a maximum dry density of 1.37 g/cm^3^ and an optimal moisture of 20.0%. As presented in Table 2, X-ray fluorescence spectroscopy (XRF) analysis indicated that the premary chemical components of PG were CaO and SO_3_, with a minor amounts of SiO_2_ and P_2_O_5_. Lime (L), procured locally, had an available CaO content exceeding 90 wt%. Commercial sodium silicate (water glass, NS) with a modulus of 3.3 was supplied Jiangsu Yannai New Material Technology Co., Ltd.

**Table 2:** Main chemical components of phosphogypsum.

Element	CaO	SO_3_	SiO_2_	P_2_O_5_	Fe_2_O_3_	Al_2_O_3_
Content (%)	29.47	26.00	3.06	0.84	0.75	0.61

Phosphogypsum (PG), lime (L) and sodium silicate (water glass, NS) were sourced as raw materials for the soil binder formulation. Lime and sodium silicate were incorporated to amend PG, so as to enhancing its performance. The binder proportion was determined via preliminary single-factor optimization tests, which showed that 12% total binder (3% lime, 1% sodium silicate by PG mass) minimized swelling and maximized strength with acceptable cost. The total binder content was fixed at 12% by mass relative to the dry soil. LSP composition (by dry soil mass): 11.52% phosphogypsum, 0.36% lime, 0.12% sodium silicate (total 12%). Within this binder, lime and sodium silicate constituted 3% and 1% of the PG mass, respectively. Both additives establish a highly alkaline environment, significantly reducing PG solubility. Furthermore, lime releases calcium ions while sodium silicate provides soluble silicates, enabling participation in subsequent hydration and pozzolanic reactions, thereby improving soil stabilization efficacy.

#### (3) Sample preparation.

The tested soil was oven-dried at 105 °C for 24 h, pulverized and sieved through a 2-mm mesh. Oven-drying at 105°C follows GB/T 50123−2019 [42] to remove free water and ensure uniform initial moisture. PG and lime were similarly processed (dried at 60 °C for 48 h), and sieved with a-0.5 mm mesh. PG and lime powders were first dry-mixed according to the designed binder ratio. This blend was then homogenized with the prepared soil. A predetermined mass of sodium silicate, calculated based on the optimal water content, was dissolved in distilled water. The sodium silicate solution was added to the solid mixture and stirred thoroughly for 5 min using a high-speed agitator.

The resulting mixture was compacted into the cylindrical molds (*Φ* 39.1 mm × *H* 80 mm), or cutting rings (*Φ* 61.8 mm × *H* 20 mm or 40 mm) at a constant displacement rate of 1 mm/min. Cylindrical specimens were demolded prior to curing, while specimens in cutting rings were cured directly within the rings. All specimens were sealed in plastic film and cured for 28 days in a standard curing room with humidity of 95 ± 2% and temperature of 22 ± 2 °C.

### 2.2 Test methods

#### (1) Cyclic drying-wetting (D-W) test.

According to ASTM D4843-88, the cyclic drying-wetting (D-W) test was conducted on the specimens curing for 28 days. Procedures constitute a complete D-W cycle were listed as follows:

① **Wetting process:** Specimens were placed on saturated porous stones within plastic containers. Deionized water was added to each container until the water level was flush with the top surface of the porous stone. Specimens were soaked for 24 h, allowing capillary uptake through their pore structure.
② **Drying process:** After finishing the wetting process, specimens were removed from the containers and dried in a forced-air oven at 45 °C for 24 h. Subsequently, specimens were taken out of the oven and cooled to ambient temperature (approximately 22 °C) prior to testing or subsequently cycling.

Specimens underwent 0, 1, 3, 5, 10, or 15 designated cycles, denoted as F0, F1, F3, F5, F10 and F15, respectively. 15 cycles correspond to 15 years of seasonal wet-dry variation in subtropical regions, simulating medium-term subgrade durability.

#### (2) Geotechnical tests.

A series of geotechnical tests, including compaction, free swell ratio, unconfined compressive strength, shear strength, consolidation and permeability tests, were carried out to assess the long-term durability of the stabilized expansive soil with lime-sodium silicate amended PG subjecting to various cycles of D-W process. Shear strength was determined via direct shear test (ZJ-2 apparatus) at 50−400 kPa normal stress, shear rate 0.02 mm/min (GB/T 50123−2019) [42]. All the above tests were performed according to Chinese standard GB/T 50123 (2019) [42].

#### (3) XRD and MIP tests.

Specimens after experiencing the specific D-W cycles were carefully cut into strips with cross-section of different size. These select samples were placed in pre-labeled aluminum boxes and freeze-dried (Alpha 1–4 LDplus; −59°C) for 24 hours. Specimens subjected to the designated D-W cycles were carefully sectioned into strips of varying cross-sectional dimensions. The selected samples were placed in pre-labeled aluminum boxes and freeze-dried (Alpha 1–4 Lplus; −59 °C) for 24 hours. Specimens subjected to the designated D-W cycles were carefully sectioned into strips of varying cross-sectional dimensions. The selected samples were placed in pre-labeled aluminum boxes and freeze-dried (Alpha 1–4 Lplus; −59 °C) for 24 hours. XRD/MIP samples were taken from the central core (1 cm below surface); 5 g for XRD, 1 cm^3^ for MIP. Samples intended for XRD analysis were pulverized in an agate mortar and passed through a 0.075-mm mesh. MIP analysis was conducted on cubic samples (approximately 1 cm^3^) using a mercury intrusion porosimeter (Macautopore IV 9600).

## 3. Results

### 3.1 Compaction characteristic

**Fig 3:**
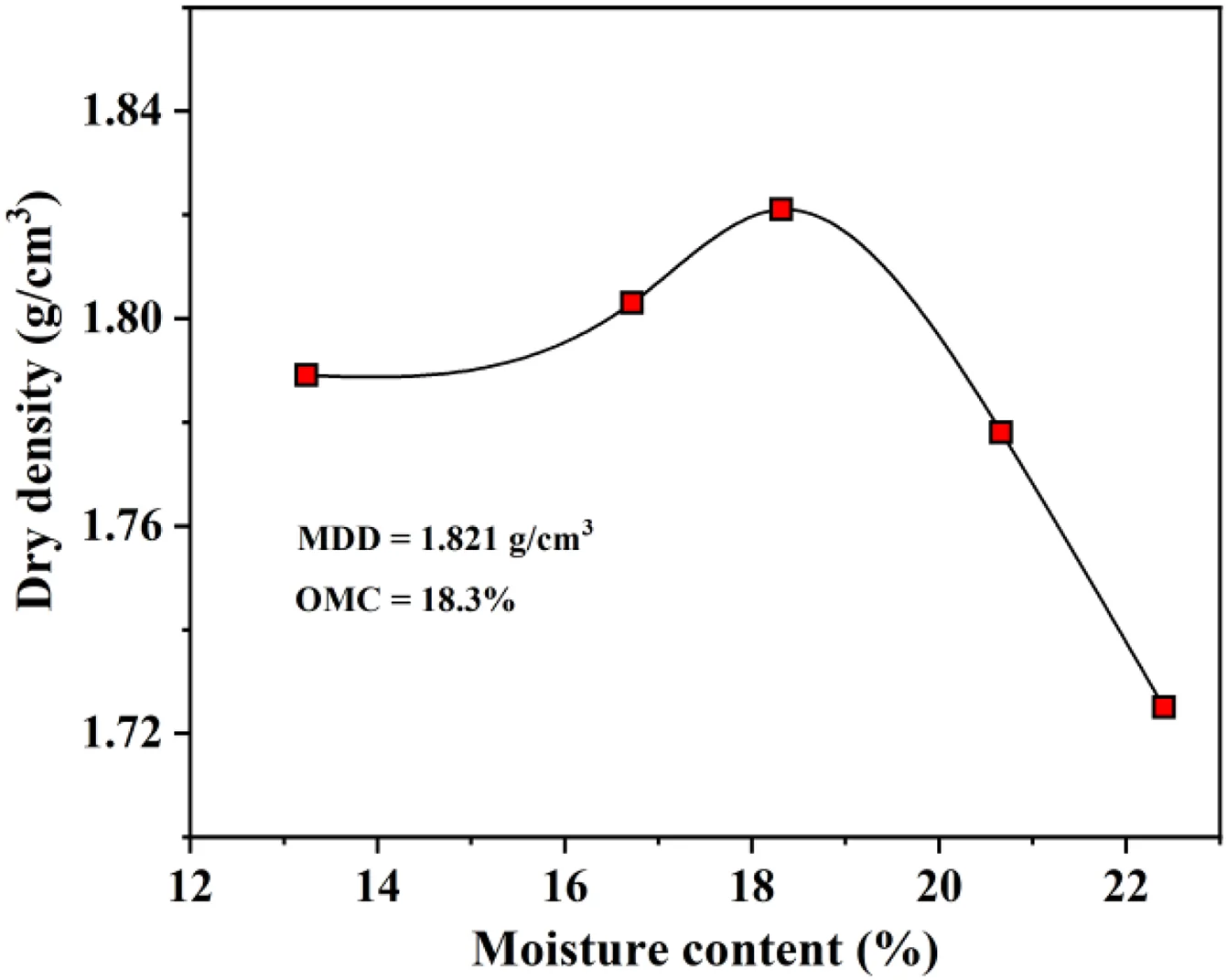
Compaction curve of the LSP stabilized expansive soil.

Fig 3 shows the compaction curve of the expansive soil stabilized with lime-sodium silicate amended PG (denoted as LSP). For the LSP stabilized soil, the maximum dry density (MDD) and optimal moisture content (OMC) were determined as 1.821 g/cm^3^ and 18.315%, respectively. Compared to the natural expansive soil, the -addition of LSP resulted in an increase in MDD while reducing the OMC.

Upon contact with water, PG dissolves, releasing a significant amount of Ca^2+^. Based on the cation exchange, Ca^2+^ substitute monovalent cations (e.g., Na^+^, K^+^) adsorbed on the surface of clay particles, thereby reducing the thickness of the diffusion double layer. Consequently, the inter-particle distance decreases, facilitating the aggregation of soil particles and leading to an increase in dry density. Correspondingly, the reduction in the thickness of diffusion double layer also diminishes the water adsorption capacity of the clay, inducing a decrease in the OMC.

Besides, the addition of lime and sodium silicate can obvious enhance the pH of the stabilized soil, and the released OH^-^ can promote the pozzolanic reactions and resulting in the generation of hydrated silicates and hydrated aluminates. These cementitious products can further enhance the density of the treated soils. Simultaneously, partial free water will transform into the crystallized water incorporating into the cementitious products, which can also reduce the OMC.

Furthermore, incorporating lime and sodium silicate significantly increases the pH of the stabilized soil. The released hydroxide ions (OH^−^) promote pozzolanic reactions, generating cementitious hydrated silicates and aluminates. These products can further densify the soil matrix, leading to an increase of the dry density. Concurrently, a portion of pore water incorporates into these cementitious phases as crystalline water, thereby reducing the optimum moisture content (OMC).

### 3.2 Free swell analysis

**Fig 4:**
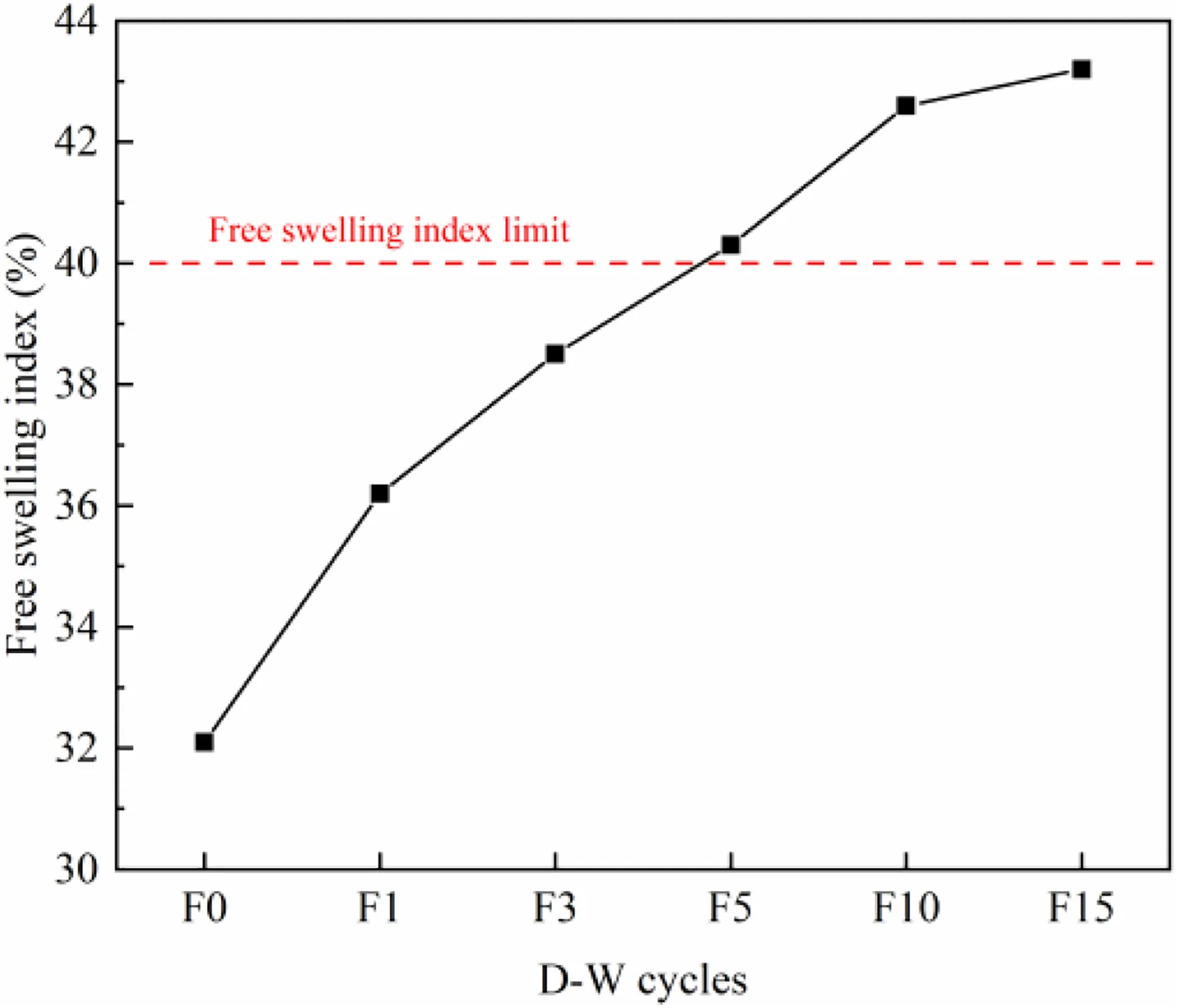
Effect of the number of D-W cycles on the free swelling index.

Fig 4 illustrates the effect of drying-wetting (D-W) cycle on the free swelling index of the stabilized soil. The free swelling index of the untreated soil was 56.8%. After stabilization with LSP, the free swelling index decreased significantly to 32.1%, indicating that the additive effectively reduced the swell potential of the expansive soil.

However, after being subjected to repeated D-W cycles, the free swelling index of the stabilized sample increased obviously. Between 0 and 10 cycles, the free swelling index increased linearly with the number of drying-wetting cycle. As the number of D-W cycles increased from 10 to 15 cycles, the free swelling index continued to rise, but with a reduced increase amplitude. Critically, when the stabilized sample was subjected to 5 cycles of D-W, the free swelling index is 40.3%, depending on which the sample was transformed to expansive soil again.

FSI increases due to PG dissolution, Ca^2+^ leaching (DDL thickening), and microcrack development.

### 3.3 Unconfined compressive strength (UCS)

**Fig 5:**
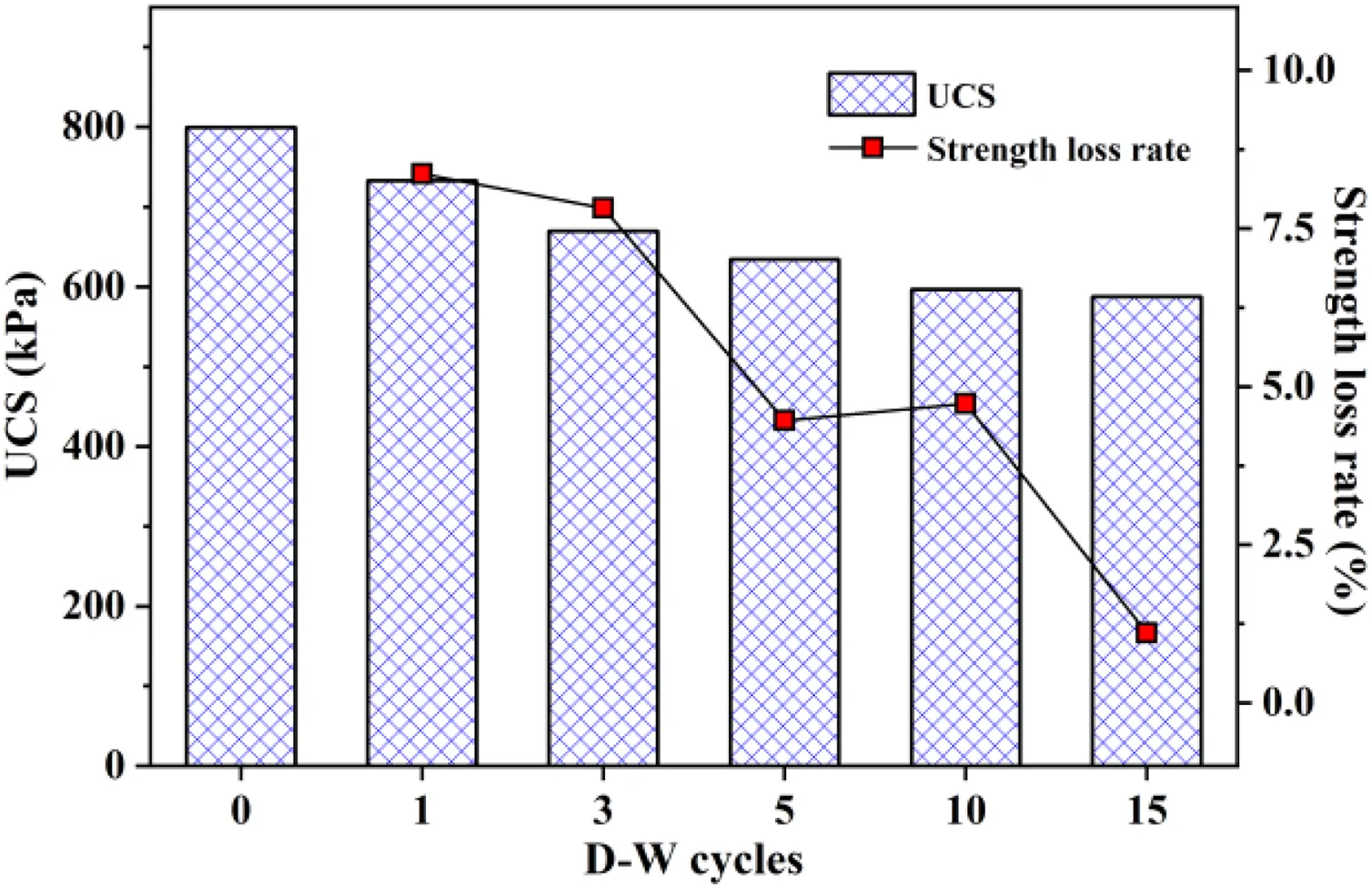
Effect of the number of D-W cycles on the UCS and strength loss rate.

Fig 5 illustrates the relationship between the UCS of the LSP stabilized specimens and the number of D-W cycles. It can be observed that the UCS decreased gradually with increasing D-W cycles.

To quantitatively evaluate the impact of D-W cycles on UCS development in LSP stabilized specimens, the UCS loss rate induced by cyclic D-W was calculated and plotted in Fig 4 as well. After 15 D-W cycles, the total UCS loss of the specimens reached 26.5%. Remarkably, 16.2% absolute loss occurred within the first 3 cycles accounted for 61.1% of this total degradation. It indicates that the initial D-W cycles inflict the most severe damage to the structural integrity of the stabilized soil. As D-W cycles increase from 3 to 5 and 5–10, UCS losses were comparable at 4.46% and 4.73%, respectively. This demonstrates progressive attenuation of the deterioration effects aroused by D-W cycles. Critically, as the number of D-W cycles increased from 10 to 15, the UCS fluctuation appeared to be slight with a strength loss of 1.1%.

### 3.4 Shear strength

**Fig 6:**
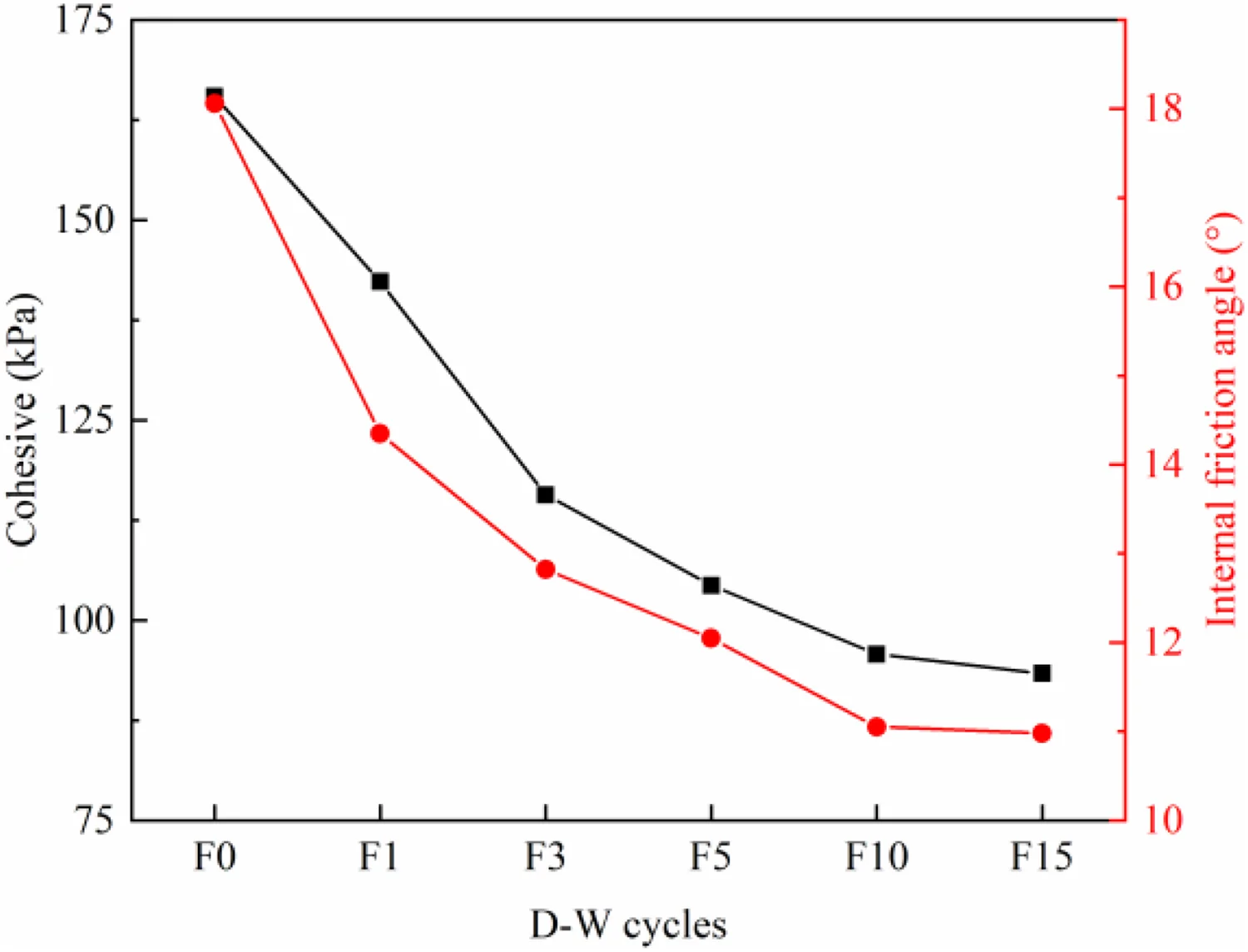
Effect the number of D-W cycles on the cohesion and internal friction angle.

Fig 6 presents the effect of D-W cycles on the cohesion and internal friction angle of the LSP stabilized samples. Friction angle was measured via direct shear test, not UCS. Both parameters decrease significantly during the initial 10 D-W cycles, and followed by a marginal reduction over the subsequent 5 cycles. The evolution of cohesion and internal friction angle induced by repeated D-W cycles followed a pattern similar to that observed for UCS.

During the wetting phase of D-W cycles, Ca^2+^ present in the pore solution leach from the sample due to the concentration gradient between its interior and the surrounding environment. This leaching reduces the Ca^2+^ concentration within the soil pore solution, leading to an increase in the thickness of the DDL and consequent swelling of the soil. Li et al. (2015) [45] emphasized that DDL played an important role on soil cohesion. The increase of DDL thickness will cause increase of the repulsion and decrease of Van der Waals’ attractive forces among the clay particles, which results in a decrease of cohesion. Concurrently, PG constituted the soil skeleton dissolves during wetting due to its inherent solubility. The voids left by PG dissolution increase the overall pore volume and weaken the soil skeleton. Consequently, the interlocking action between particles diminishes, resulting in a reduction of both cohesion and internal friction angle.

During the drying phase of D-W cycles, water evaporation causes decrease in the thickness of DDL surrounding clay particles. This reduction promotes aggregation of solid particles while simultaneously increasing the spacing between aggregates. Critically, these gaps between aggregates undergo progressive enlargement with repeated D-W cycles, ultimately evolving into fissures. The development of fissures induced by D-W cycles imposes detrimental impacts on the shear strength of the stabilized samples.

### 3.5 Compressibility

**Fig 7:**
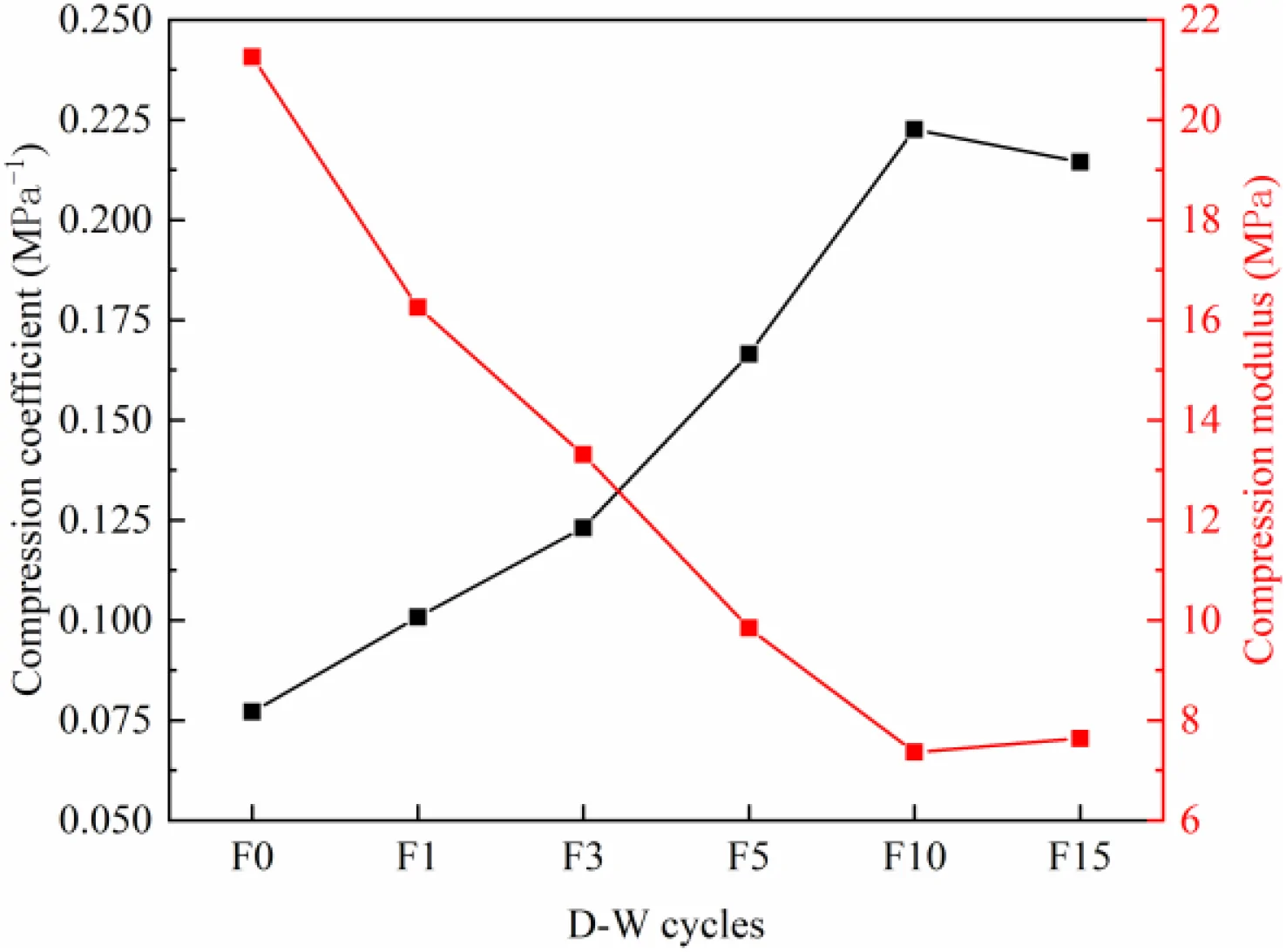
Effect of the number of D-W cycles on the compressibility.

Compression coefficient and compression modulus of the LSP stabilized samples subjected to various D-W cycles were plotted in Fig 7. Compression modulus (Es) = Δσ/Δε, representing soil stiffness under vertical loading. Units: compression coefficient (MPa^-1^), modulus (MPa). During the first 10 cycles of D-W, the compression coefficient of the specimens progressively increased, while the compression modulus gradually decreased. However, when the number of D-W cycles increased from 10 to 15, the compression coefficient exhibited a marginal decrease, and the compression modulus showed a slight rebound.

Notably, the compression coefficient and compression modulus exhibit significant degradation during the initial 10 D-W cycles. Successive D-W cycles enhance the dissolution of PG and increase the porosity of the specimens, resulting in the increase of compressibility. Besides, reduction in the thickness of DDL due to Ca^2+^ leaching out of specimens can also make the specimens easier to be compressed.

### 3.6 Permeability

**Fig 8:**
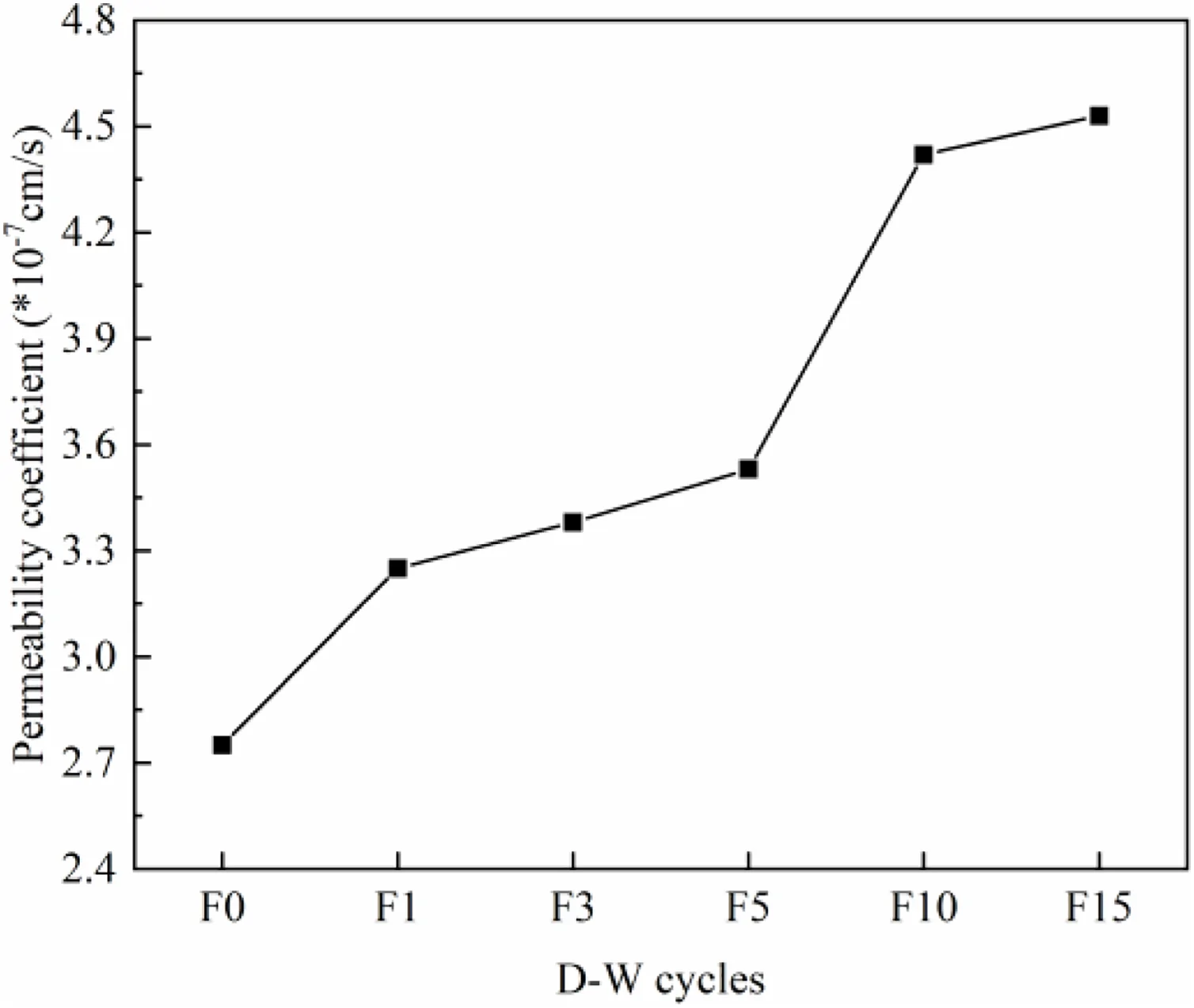
Relationship between the permeability coefficient and the number of D-W cycles.

**Fig 9:**
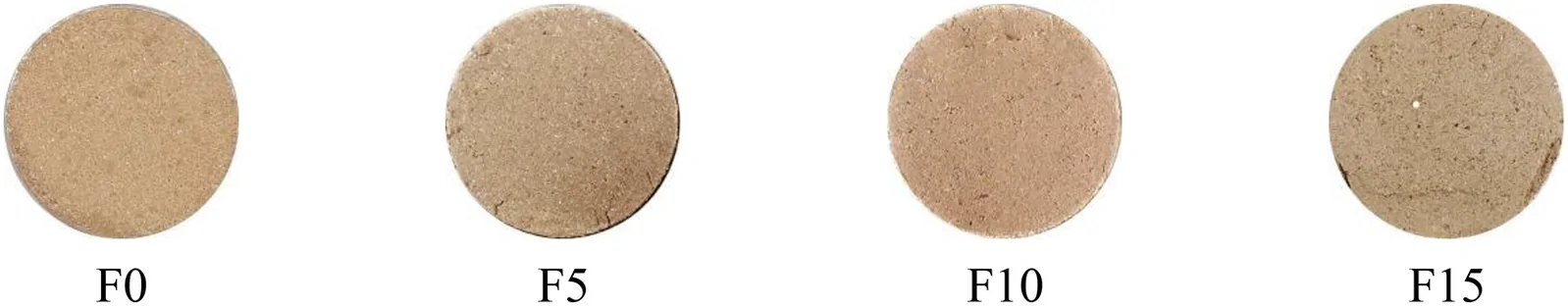
Effect of the number of D-W cycles on the surface cracks of specimens.

Fig 8 illustrates the effects of D-W cycles on the permeability of the LSP stabilized soil. It can be observed that the permeability coefficient had a negative relation with the D-W cycles. As the number of D-W cycles increased from 0 to 15, the permeability coefficient increased from 2.75 × 10^−7^ cm/s to 4.53 × 10^−7^ cm/s, a 1.65-fold enhancement. Permeability increases arise from enhanced connectivity of micropores and mesopores due to PG dissolution, not from large connecting cracks. No visible desiccation cracks were observed on specimen surfaces after 15 cycles; only microcracks (<0.1 mm) detected by MIP (Effect of the number of D-W cycles on the surface cracks of specimens are shown in Fig 9).

## 4. Discussion

### 4.1 XRD analysis

**Fig 10:**
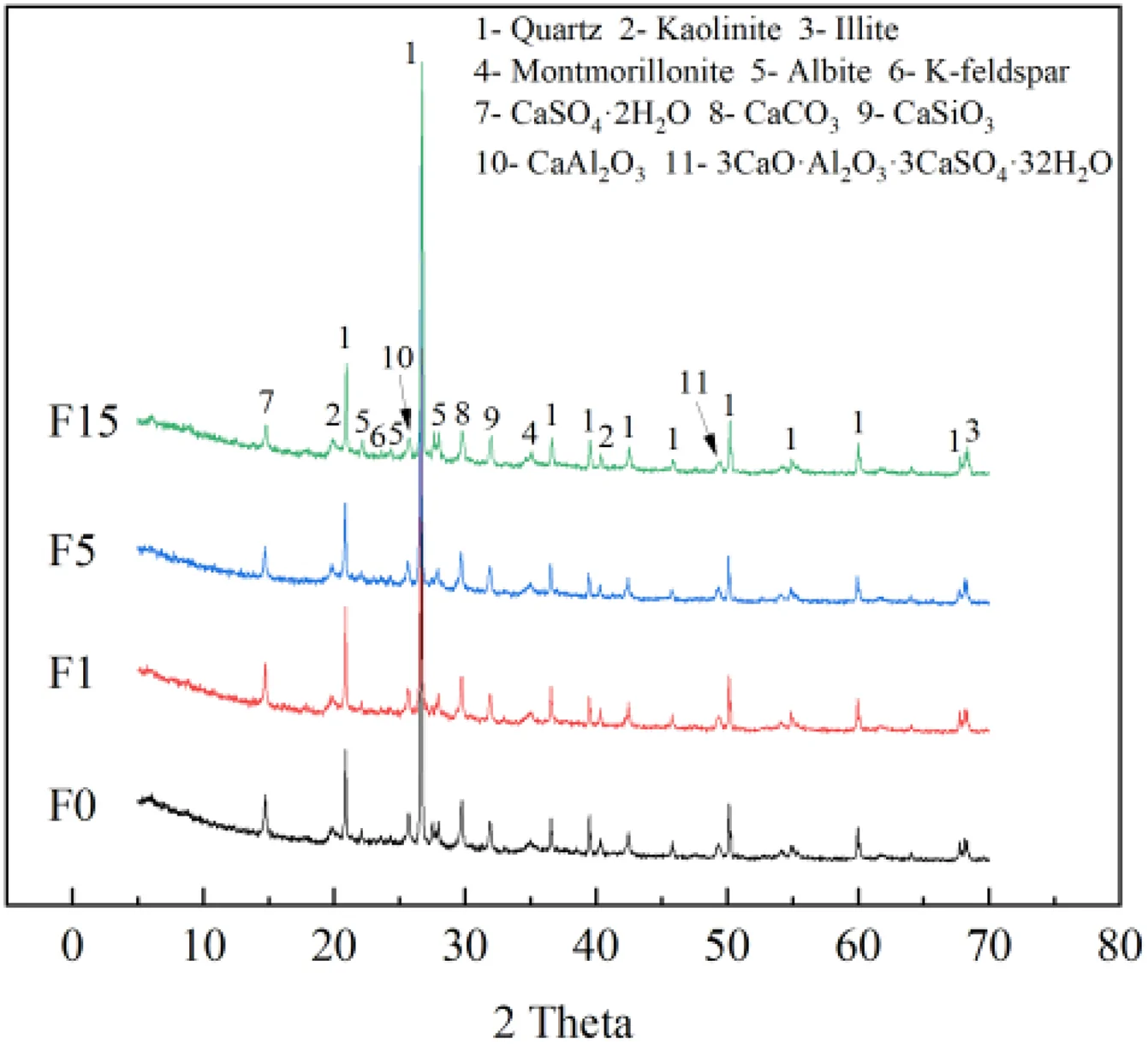
XRD patterns of the specimens subjected to D-W cycles.

The mineral compositions of specimens before and after subjecting to D-W cycles were analyzed by XRD, as shown in Fig 10. Compared with the untreated expansive soil, several new minerals, including calcium silicate hydrate (C-S-H), calcium aluminate hydrate (C-A-H), ettringite, gypsum (CaSO_4_·2H_2_O), and calcite (CaCO_3_), were detected in the LSP stabilized specimens. Formation of these cementitious products fill the soil pores and enhance the density of specimens, improving the engineering behavior of expansive soil. After subjecting to D-W cycles, the primary mineral phases remained chemically stable. However, the peak intensity of CaSO_4_ ∙ 2H_2_O detected at 2θ of 15° decreased with increasing the D-W cycles. This demonstrates that several quantity of CaSO_4_ ∙ 2H_2_O sourced from PG dissolves during the successive D-W cycles. It can be concluded that deterioration of the engineering behaviors including free swelling index, UCS, shear strength, compressibility and permeability can predominantly owing to the dissolution of PG.

### 4.2 MIP

**Fig 11:**
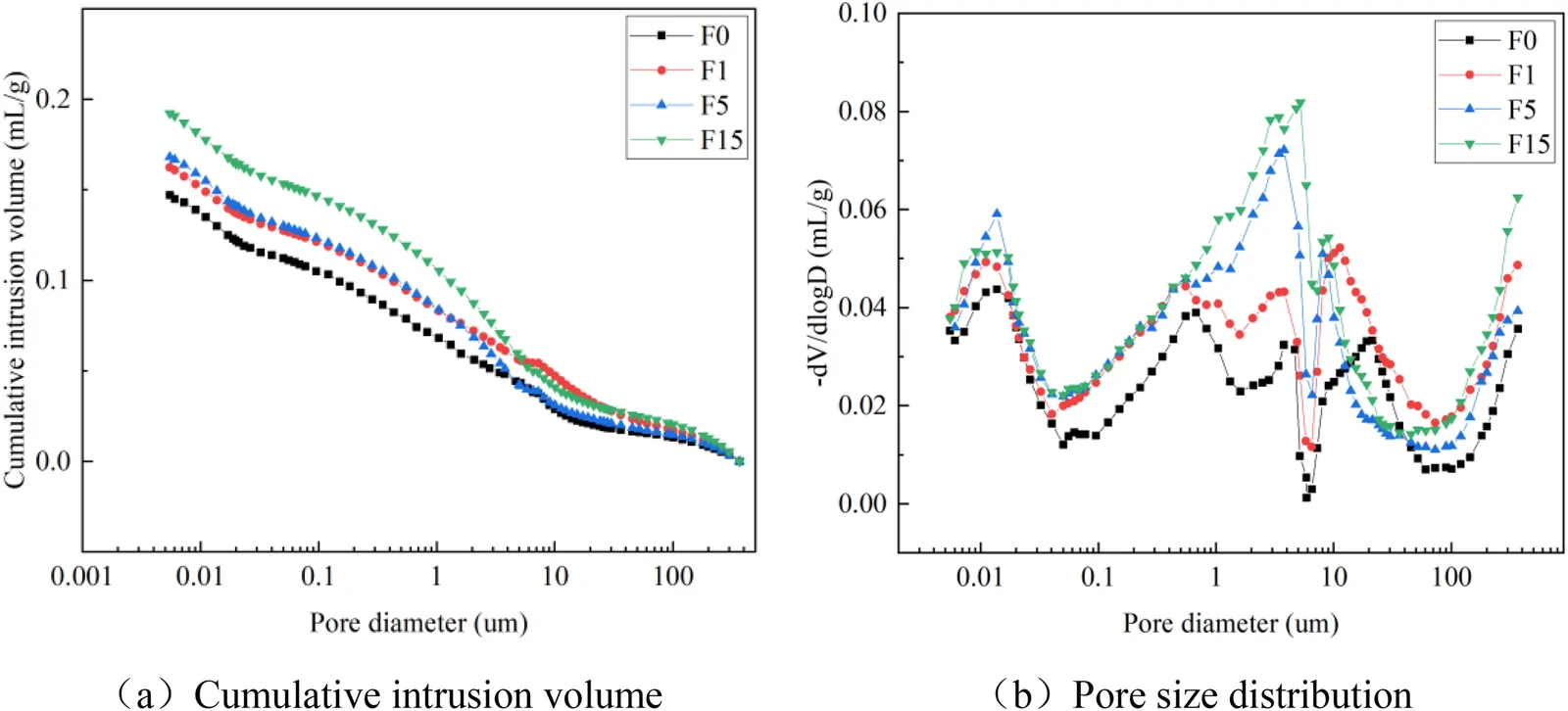
Pore size distribution characteristics of LSP stabilized specimens under cyclic D-W conditions.

The relation curves of the pore size diameter and the corresponding differential pore volume, namely, pore size distribution curves of the specimens experiencing different cycles of D-W were shown in Fig 11.

Fig 9(a) shows the relationships between the cumulative intrusion volume and pore diameter of the specimens subjected to 0, 1, 5 and 15 cycles of D-W. With increasing the number of D-W cycles, the cumulative intrusion volume increased evidently. It is proved that D-W cycles enlarge the porosity of the stabilized soils, which leads to the deterioration of the engineering performance.

Depending on the pore size distribution curve shown in Fig 9(b), pores contained in the stabilized soil can be classified as four categories: ① micropore with pore diameter smaller than 0.05 μm, ② small pore with diameter of 0.05 ~ 1.5μm, ③ medium pore with diameter of 1.5 ~ 6.0 μm, and ④ large pore with diameter lager than 6 μm. Increasing the cycles of D-W gives rise to an increase in the amount of micropores. This intensifies that Ca^2+^ release out of specimens resulted from the cyclic D-W can cause the reduction of the thickness of DDL surrounding the soil particles. Thus, the spacing between soil particles is enlarged, resulting in an increase of micropores. DDL thickness changes are supported by XRD basal spacing (montmorillonite) and MIP pore size data. Compared to the specimens without D-W cycles, experiencing 1 cycle of D-W causes an obvious increase of the large pores. This indicates that several cracks formed during the first D-W cycle, which causes a significant deterioration of the engineering properties of the specimens. As the number of D-W cycles increases continuously, the small pores disappear, which were transformed into medium pores under successive D-W cycles. While the amount of large pores was maintained at approximately constant. Total pore volume increases, and micropore connectivity improves, even if mean pore diameter slightly decreases. On this basis, the continuous deterioration on the engineering performance of specimens after the number of D-W cycles exceeds 5 can be attributed to the increase of the number of medium pores.

### 4.3 Stabilization mechanisms based on LSP

Stabilization mechanisms involved in the LSP stabilized expansive soil include physical filling, cation exchange, lime hydration, pozzolanic reactions and geopolymerization. Cation exchange occurs between the Ca^2+^ released from PG dissolution and lime hydration and monovalent cations (e.g., Na^+^, K^+^) within the DDL. As a result, the DDL thickness decreases, leading to a reduction in the swelling-shrinkage potential of expansive soil. Furthermore, the fine-grained nature of PG particles enables them to act as a filler, increasing the dry density of the expansive soil. Lime hydration and sodium silicate decomposition elevate the pH of the stabilized matrix. This alkaline environment inhibits PG dissolution and enhances specimen stability under humid conditions. The Ca^2+^ and OH^-^ released during lime hydration react with ${\text{SO}}_{\text{4}}^{\text{2}-}$ sourced from PG and aluminates derived from the expansive soil to form ettringite (AFt). Additionally, geopolymerization between sodium silicate and clay minerals generates calcium (alumino)silicate hydrate (C-(A)-S-H), which also contributes to the improved performance of the stabilized soils. However, given the relatively low contents of lime and sodium silicate in the LSP mixture, the formation of AFt and C-(A)-S-H plays a minor role in the overall improvement of the expansive soil.

### 4.4 Deterioration mechanism associated with D-W cycles

XRD and MIP analyses, presented in Fig 10 and Fig 11, respectively, indicate that partial dissolution and leaching of PG occurred under D-W cycles, resulting in the development of micro-cracks and an increased pore volume within the specimens. The formation of these micro-cracks appears to be the primary mechanism responsible for the deterioration of the engineering properties of specimens. Besides PG dissolution, minor decalcification of C-S-H and AFt occurs during wetting, further weakening the microstructure.

Strength loss and microstructural evolution under D-W cycles are consistent with recent durability studies [38,46].

## 5. Conclusions

This paper investigates the engineering performance of LSP stabilized expansive soil under cyclic D-W condition. Besides, the stabilization mechanism based on LSP and deterioration mechanism involved in D-W cycles are both discussed deeply. The main conclusions obtained by the present study are listed as follows:

(1) The maximum dry density increases while the optimal moisture content decreases after the expansive soil is stabilized with 12% LSP, and the swelling-shrinkage potential is effectively weakened as well.
(2) D-W cycles impose obviously detrimental impacts on the engineering performance of the LSP stabilized soil, including free swelling index, UCS, shear strength, compressibility and permeability.
(3) Physical filling by PG powder plays an important role on the improvement of the soil engineering properties.
(4) PG dissolution and micro-cracks development appear to be responsible for the deterioration of the stabilized soil subjected to D-W cycles, while the latter may be dominant.
(5) Environmental implications: LSP enables full phosphogypsum valorization with low leaching risk and 30–40% lower carbon emissions than conventional binders.
